# Macrophages induce “budding” in aggressive human colon cancer subtypes by protease-mediated disruption of tight junctions

**DOI:** 10.18632/oncotarget.24626

**Published:** 2018-04-13

**Authors:** Kari Trumpi, Nicola Frenkel, Timo Peters, Nicoline M. Korthagen, Jennifer M.J. Jongen, Daniëlle Raats, Helma van Grevenstein, Yara Backes, Leon M. Moons, Miangela M. Lacle, Jan Koster, Danny Zwijnenburg, Inne H.M. Borel Rinkes, Onno Kranenburg

**Affiliations:** ^1^ UMC Utrecht Cancer Center, University Medical Center Utrecht, Utrecht, The Netherlands; ^2^ Orthopedics, University Medical Center Utrecht, Utrecht, The Netherlands; ^3^ Department of Equine Sciences, Faculty of Veterinary Medicine, Utrecht University, Utrecht, The Netherlands; ^4^ Gastroenterology & Hepatology, University Medical Center Utrecht, Utrecht, The Netherlands; ^5^ Pathology, University Medical Center Utrecht, Utrecht, The Netherlands; ^6^ Department of Oncogenomics, Academic Medical Center, University of Amsterdam, Amsterdam, The Netherlands

**Keywords:** colorectal, budding, metastasis, consensus molecular subtypes, matrix metalloprotease

## Abstract

Primary human colorectal tumors with a high stromal content have an increased capacity to metastasize. Cancer-associated fibroblasts (CAFs) promote metastasis, but the contribution of other stromal cell types is unclear. Here we searched for additional stromal cell types that contribute to aggressive tumor cell behavior. By making use of the ‘immunome compendium’—a collection of gene signatures reflecting the presence of specific immune cell-types—we show that macrophage signatures are most strongly associated with a high CAF content and with poor prognosis in multiple large cohorts of primary tumors and liver metastases. Co-culturing macrophages with patient-derived colonospheres promoted ‘budding’ of small clusters of tumor cells from the bulk. Immunohistochemistry showed that budding tumor clusters in stroma-rich areas of T1 colorectal carcinomas were surrounded by macrophages. *In vitro* budding was accompanied by reduced levels of the tight junction protein occludin, but *OCLN* mRNA levels did not change, nor did markers of epithelial mesenchymal transition. Budding was accompanied by nuclear accumulation of β-catenin, which was also observed in budding tumor cell clusters *in situ*. The NFκB inhibitor Sanguinarine resulted in a decrease in MMP7 protein expression and both NFκB inhibitor Sanguinarine and MMP inhibitor Batimastat prevented occludin degradation and budding.

We conclude that macrophages contribute to the aggressive nature of stroma-rich colon tumors by promoting an MMP-dependent pathway that operates in parallel to classical EMT and leads to tight junction disruption.

## INTRODUCTION

Traditional staging of human colorectal cancer is not sufficiently informative to predict the formation of distant metastases in patients with localized disease. The histopathological features that are currently used as indicators of potentially aggressive disease include lymphovascular invasion, a poor differentiation grade, and—in T1 colorectal carcinomas—deep submucosal invasion (≥1,000 μm) and ‘tumor budding’, *i.e.* the presence of small clusters of tumor cells in the stroma at the invasive front [1, 2]. Large scale DNA analysis has so far failed to identify (patterns of) mutations in specific genes that are associated with metastasis or with histopathological features.

Gene expression profiling and RNA sequence analysis shows that recurrent patterns of gene expression can be used to define a limited number of ‘molecular subtypes’ in CRC [3–8]. These subtypes show marked differences in biological characteristics as well as in a patient's prognosis. Currently, four consensus molecular subtypes (CMS1-4) are recognized: CMS1 (MSI, Microsatellite Instability, 14% of CRC cases), hypermutated, strong immune activation; CMS2 (Canonical, 37%), epithelial, chromosomally unstable, marked WNT and MYC signaling activation; CMS3 (Metabolic, 13%), epithelial, evident metabolic dysregulation; and CMS4 (Mesenchymal, 23%), prominent transforming growth factor β activation, stromal invasion, and angiogenesis [3–9]. The most aggressive subtype (CMS4) has the worst prognosis of all the subtypes as it shows greater tendency to form distant metastases as well as an association with chemotherapy-resistance. CMS4 is named the mesenchymal subtype as it shows atypical expression of mesenchymal genes [9], which is largely due to a high stromal content in these tumors [10, 11]. Stromal fibroblasts play a causative role in the metastatic process [12], but the potential contribution of other non-cancer cells to the aggressive behavior of stroma-rich CMS4 tumors is unknown. The tumor microenvironment contains many different cell types, including immune cells. In general, systemic and regional inflammatory responses can play an important role in the pathogenesis of cancer and metastatic tumor progression [13, 14]. Indeed, the CMS4 subtype also shows signs of infiltration by immune cells [15], but how and whether this is related to the aggressive behavior of these tumors is not clear. Tumor-infiltrating immune cells can have anti- or pro-tumorigenic effects. For instance, tumors with high levels of infiltrating T cells (*i.e.* a high ‘immunoscore’) have a lower chance of distant metastasis [12]. On the other hand, different types of immune cells have also been shown to release various proinflammatory, proangiogenic and prometastatic mediators [16–18]. One type of immune cell showing this type of dichotomy of function is the tumor-associated macrophages (TAMs), which are also a major component of the immune-infiltrate of most tumors [19, 20]. Depending on signals from the tumor microenvironment, TAMs can polarize towards a tumor-suppressing M1 phenotype (fostering a T_H1_ response and the generation of anti-tumor immunity) or towards a tumor-promoting M2 phenotype [21]. The literature on the role of TAMs in CRC is ambivalent, with studies showing a tumor promoting function [22] and studies showing a tumor suppressive function [23].

The presence of immune cells in human tumor tissue is traditionally analyzed by immunohistochemistry using specific immune cell markers. In addition, RNA profiles of unsegregated tumor tissue can also be used to infer the presence of immune cell types in tumors. Highly restricted gene expression signatures in specific immune cell types form the basis for such analyses. A collection of such signatures, the ‘immunome compendium’, can be used to interrogate the immune landscape of human CRC and other cancers [24].

Aside from the CMS classification, another important adverse prognostic factor that has been well-established recently is the migration of single tumor cells or clusters at the invasive front of the tumor bulk [25–28]. This is also known as tumor budding. While tumor budding has been described in other gastrointestinal cancers such as pancreatic and esophageal carcinoma, it is most extensively studies in CRC. Tumor budding is regarded as an independent predictor of lymph node and distant metastases, recurrence and survival. While the underlying mechanisms for this phenomenon remain unclear, the migration of tumor cells is thought to represent a form of epithelial-to-mesenchymal transition (EMT) [25–29].

In the present manuscript, we have used the immunome compendium to show that macrophage signatures are highly expressed in aggressive subtypes of primary CRC and liver metastases (LM). Moreover, co-culture experiments reveal that macrophages at the tumor-stroma interface appear to trigger a ‘tumor budding-type’ invasion pathway. The underlying mechanism involved matrix metalloproteases (MMP)-mediated degradation of tight junctions. We demonstrate the existence of a macrophage- and MMP-dependent budding-type invasion pathway that operates independently of classical EMT in aggressive colorectal cancer, and describe an *in vitro* system to study this phenomenon.

## RESULTS

### High expression of macrophage signatures in aggressive subtypes of primary CRC and liver metastases

A high content of cancer-associated fibroblasts (CAF) contributes extensively to the mesenchymal phenotype of aggressive colon cancer [10, 11, 30]. The co-expression of mesenchymal signatures with signatures reflecting inflammation [15] suggests that certain immune cells may be enriched in stroma-high colon tumors and that, if this is indeed the case, they may contribute to their aggressive behavior. To test this hypothesis, we made use of the ‘immunome compendium’, a collection of gene sets reflecting the presence of specific immune cells that can be used to investigate the immune landscape of (colon) tumors [24]. We first tested the correlation of each specific immune cell signature with a signature reflecting high CAF content [31] in two large primary colon cancer cohorts and a liver metastasis cohort. In all three cohorts the signature reflecting the presence of macrophages was most strongly correlated with the CAF signature (Table 1).

**Table 1:** Immune cell signature expression in relation to stroma and consensus molecular subtypes

Table 1	PRIMARY		LM	PRIMARY		LM	PRIMARY		LM	PRIMARY		LM	PRIMARY		LM
	CIT-566	MVRM-345	LM-119	CIT-566	MVRM-345	LM-119	CIT-566	MVRM-345	LM-119	CIT-566	MVRM-345	LM-119	CIT-566	MVRM-345	LM-119
**Macrophages**	0,88	0,862	0,88	0,533	0,448	n/a	-0,461	-0,4	-0,405	-0,205	-0,163	0,076	0,775	0,767	0,793
**T-helper 1**	0,63	0,511	0,75	0,491	0,383	n/a	-0,453	-0,33	-0,199	-0,23	-0,191	0,327	0,469	0,341	0,74
**Neutrophils**	0,63	0,604	0,58	0,349	0,293	n/a	-0,42	-0,43	-0,412	-0,15	-0,108	0,056	0,483	0,417	0,379
**Dendritic cells, Immature**	0,62	0,506	0,82	0,212	-0,021	n/a	-0,351	-0,26	-0,328	0,158	0,104	0,098	0,492	0,427	0,682
**T-effector memory**	0,56	0,435	0,53	0,254	-0,047	n/a	-0,147	0,02	0,233	-0,349	-0,194	0,079	0,526	0,441	0,576
**Dendritic cells**	0,54	0,584	0,71	0,122	0,072	n/a	-0,29	-0,26	-0,504	-0,003	-0,042	-0,02	0,383	0,425	0,55
**Mast cells**	0,53	0,465	0,68	0,019	-0,1	n/a	-0,304	-0,35	-0,255	0,186	0,074	0,164	0,504	0,472	0,588
**T-follicular helper**	0,4	0,249	0,44	0,184	0,002	n/a	-0,125	-0,06	0,127	0,123	0,279	0,214	0,301	0,135	0,457
**B cells**	0,39	0,267	0,61	0,073	0,014	n/a	-0,153	-0,16	-0,244	0,098	0,282	-0,007	0,251	0,154	0,469
**T-CD8**	0,38	0,295	0,4	0,426	0,44	n/a	-0,286	-0,14	0,171	-0,003	0,092	0,082	0,27	0,151	0,447
**Eosinophils**	0,26	0,39	0,26	0,028	-0,135	n/a	-0,087	-0,03	0,24	0,132	0,074	0,31	0,255	0,367	0,246
**T gamma delta**	0,25	0,469	0,6	-0,052	-0,093	n/a	-0,207	-0,34	-0,609	0,111	0,055	-0,181	0,316	0,294	0,421
**Natural Killer cells**	0,22	0,147	0,53	-0,148	0,051	n/a	-0,169	-0,07	0,03	-0,084	-0,043	0,262	0,216	0,233	0,551
**T central memory**	0,19	0,172	0,19	0,248	0,095	n/a	0,101	0,14	0,424	-0,1	-0,051	0,162	0,149	0,155	0,229
**Dendritic cells, activated**	0,18	0,11	0,43	0,391	0,446	n/a	-0,294	-0,31	-0,031	-0,038	-0,049	0,172	0	-0,09	0,352
**T-helper 2**	-0,08	-0,161	0,13	0,658	0,673	n/a	-0,158	-0,21	0,245	0,09	0,234	0,54	-0,16	-0,227	0,247
	**STROMA**			**CMS1**			**CMS2**			**CMS3**			**CMS4**		

Expression of the indicated immune compendium signatures was analyzed in large cohorts of primary colorectal tumors (CIT-566, MVRM-345) and liver metastases (LM-119). For each of the signatures used meta-gene values were generated in R2 (http://r2.amc.nl). For each signature the meta-gene expression values for all tumors in the cohort were stored as a separate track. These tracks were subsequently compared 1 by 1 in exactly the same manner as one would compare the expression of two genes in a tumor cohort. One way analysis of variance (ANOVA) was then used to assess significance of the correlation and xy plots were generated for all track-track correlations. Table 1 shows the correlation R-values belonging to each xy-plot. Two examples of such xy plots are shown in Supplementary Figure 1. The accompanying p-values (–LOG10-transforemd) are also provided in Supplementary Figure 1. The strength of the correlation is color coded from negative (blue, -1) to positive (red, +1).

**Figure 1:**
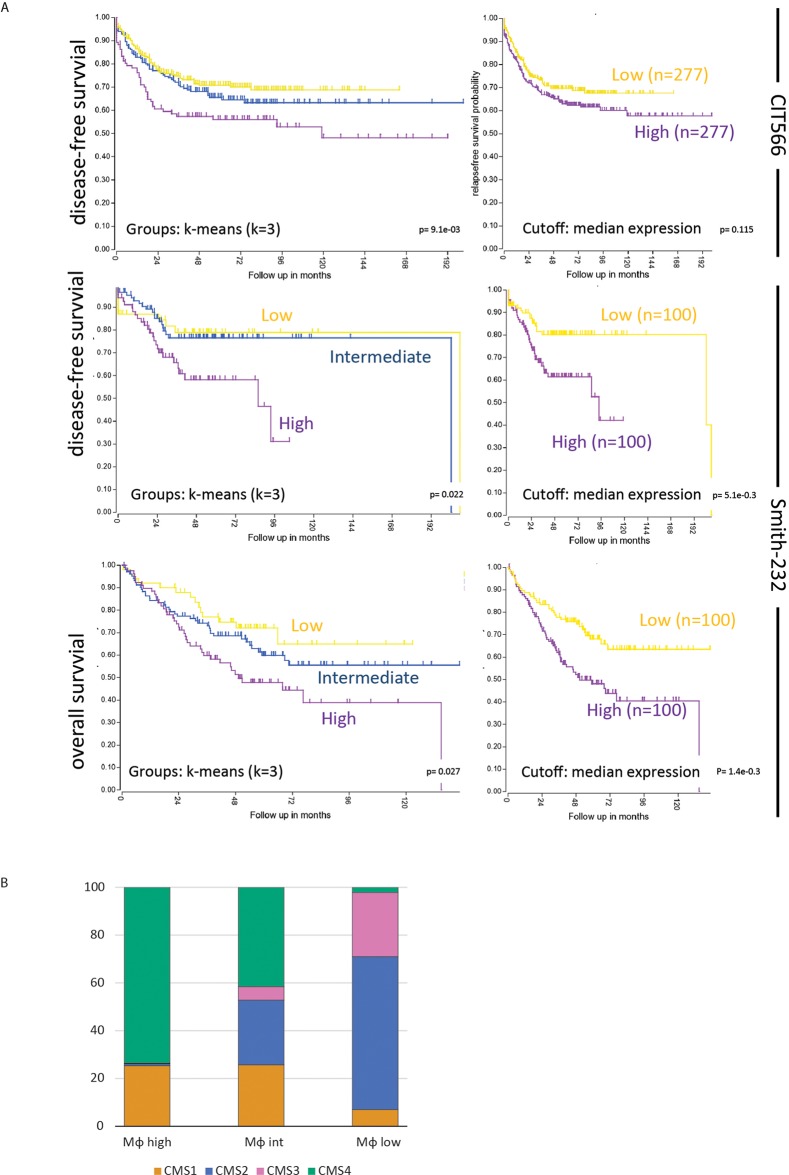
A macrophage signature identifies a poor prognosis subgroup of primary human colorectal tumors **(A)** The macrophage signature of the immune compendium was used to cluster the tumors of the CIT566 cohort into three groups by k-means clustering (http://r2.amc.nl): Macrophage high (n=142), intermediate (n=264) and low (n=396). The Kaplan Meier curve shows the differences in disease-free survival of the three subgroups. The inset shows the signature expression levels in the three subgroups. Moreover, by generating single meta-gene values of the multi-gene macrophage signature and taking the median expression of those meta-gene values as a cutoff, two equally sized groups of tumors were created in both the CIT566 and Smith cohorts. These tumors showed significant differences in disease-free and overall survival, similar to the k-means clustering. Characteristics of these cohorts shown in Supplementary Figure 1C. **(B)** Bar graphs showing the contribution of the four CMS subtypes to macrophage-high, -intermediate and -low subgroups in the CMS-3232 cohort. The CMS classification was derived from Guinney *et al.* [9].

**Figure 2:**
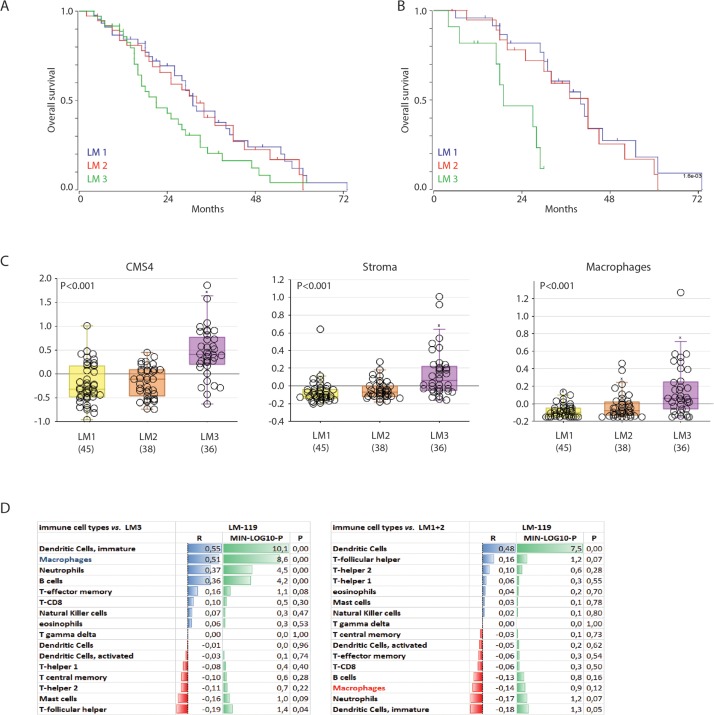
A poor prognosis subgroup of colorectal liver metastases is characterized by high expression of a macrophage signature **(A)** Gene expression profiles of 119 colorectal liver metastases were used for unsupervised clustering, yielding three ‘molecular subtypes’ (LM1-3). The figure shows Kaplan Meier curves of LM1-3 (log rank test p<0.001). **(B)** Survival curve of the chemo-naive patients (n=55), log rank test p<0.001. **(C)** Box-whisker plots showing the gene signature expression of cms4, stroma and macrophage signatures in the LM subgroups (all tested with one-way ANOVA, p<0.001). **(D)** Correlation of gene signatures corresponding with cell types in the tumor microenvironment and LM3, p-values are depicted as a – log 10 scale per R-value.

Efforts to classify human primary colon tumors into ‘molecular subtypes’ have resulted in a classification system comprising 4 Consensus Molecular Subtypes (CMS1-4) [9]. Of these subtypes, CMS4 is characterized by a high stromal content and a poor prognosis. We found that the macrophage signature was most strongly correlated with the classifier genes that positively identify CMS4 (Table 1). In line with this, the macrophage signature identified a poor-prognosis subgroup of primary human colorectal tumors (Figure 1A). The major contributor in this macrophage signature poor-prognosis subgroup is the CMS4 subtype (Figure 1B). The classification studies have so far been limited to primary CRC. To investigate the existence of molecular subtypes in colorectal liver metastases we analyzed gene expression data of a cohort of 119 liver metastases [32] by unsupervised clustering. This generated three independent subgroups (LM1-3) of which one (LM3) had a poorer prognosis when compared to the two other subgroups (p<0.001; Figure 2A). The difference in overall survival was even more pronounced when restricting the analysis to chemotherapy naïve patients (p<0.001; Figure 2B). Interestingly, the signature genes identifying CAFs, macrophages and CMS4 were significantly enriched in LM3 indicating that LM3 resembles CMS4 (Figure 2C). Moreover, analysis of the immunome compendium in relation to the LM subtypes revealed that macrophages were highly enriched in LM3, as they are in CMS4, while their presence was negatively correlated with LM1 and LM2 (Figure 2D).

### M2-macrophages cause tumor cell budding from the colonosphere bulk

**Figure 3:**
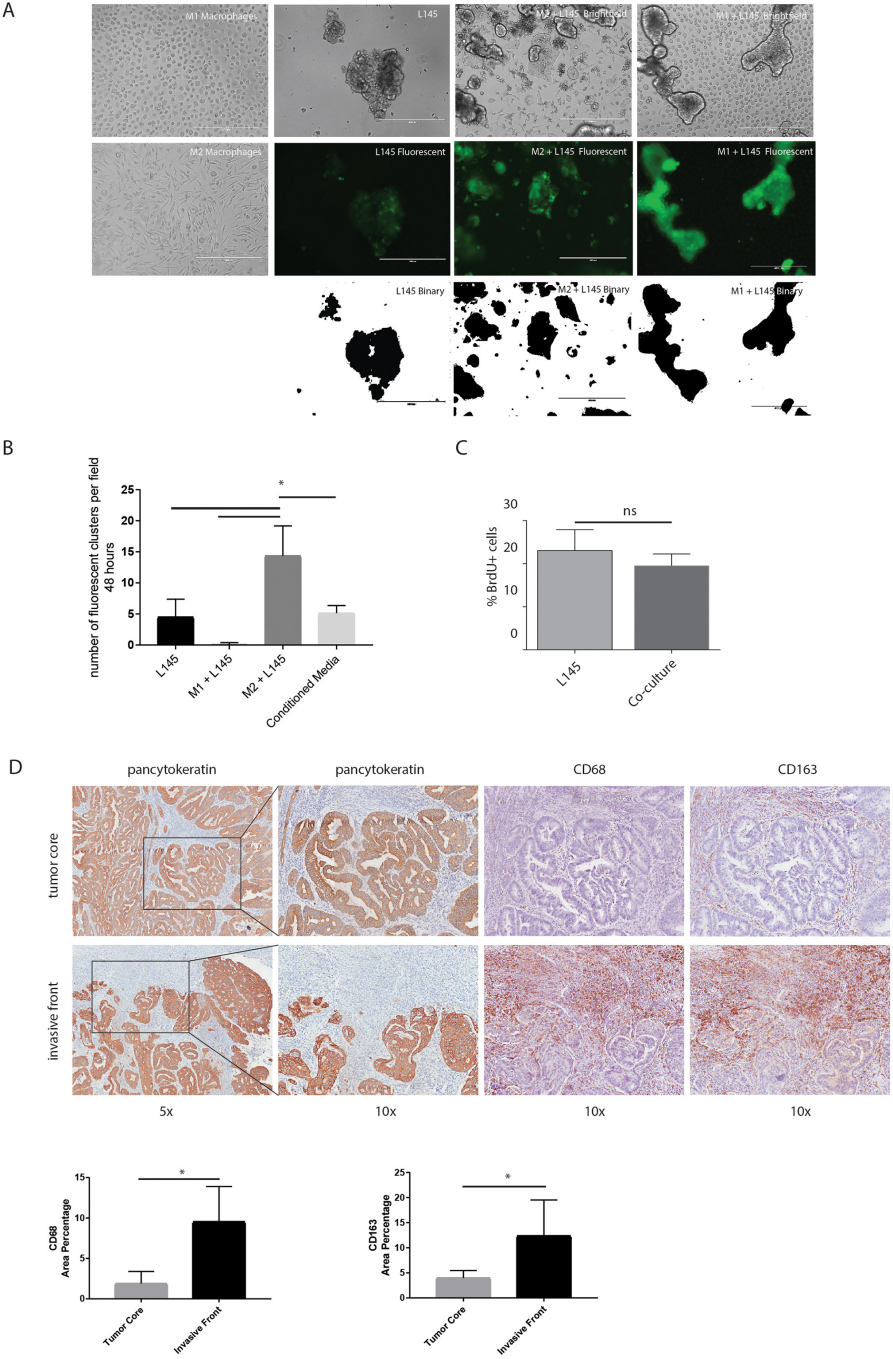
M2 macrophages cause tumor cell budding from the colonosphere bulk **(A)** Representative images of bright field microscopy of the mono-cultures and co-cultures are shown in the upper panel. Green fluorescent images to identify tumor cells are shown in the middle panel, binarized images of the fluorescence are shown in the lower panel, 10x magnification on EVOS microscope; scale bars indicate 400 μm. **(B)** Adherence assay showing the number of small cell clusters (≤5 cells) per condition; one-way ANOVA, p=<0.0001. **(C)** Proliferation of colonospheres measured on FACS Caliber after a BrdU pulse chase experiment. **(D)** Representative bright field microscopy images of the invasive front and the tumor core of T1-tumors using the immunohistochemical stainings Pan-cytokeratin (tumor cells), CD68 and CD163 (macrophages). Quantification of immunohistochemical stainings, depicted as percentage of area on the left CD68 and on the right CD163; n=8 different tumors, one image field per tumor for the invasive field and one field for the tumor core; bar graphs show mean and SD, paired t-test, p=0.0006 and p=0,0052 respectively.

To study the influence of macrophages on colorectal tumor cells, we set up a co-culture system for macrophages and patient-derived colonospheres (L145 and L169; derived as described before by Emmink *et al* [33]). Monocytes from healthy donors were isolated and differentiated into M2-macrophages via IL-10 stimulation (Supplementary Figure 2). Co-culturing M2 macrophages with colonospheres in suspension for 48 hours caused adherence of the tumor cells to the dish and induced tumor cell spreading. Moreover, small tumor cell clusters detached from the colonosphere bulk. This tumor cell behavior was not observed when co-culturing the colonospheres with anti-tumor M1 macrophages (Figure 3A). To quantify this tumor cell phenotype, we performed adhesion assays showing a 3.2-fold macrophage-induced increase in the adherence of small tumor clusters (≤ 5 cells) after 48 hours co-culturing respectively (p<0.0001). This effect was not reproduced by M2-conditioned media, suggesting the requirement for direct cell-cell interaction. To examine whether the macrophage-induced increase in ‘budding’ was due to a change in proliferation, we performed proliferation assays which showed no significant difference in proliferation between the spheroid cultures and the co-cultures (Figure 3C). To further study a potential association of macrophages with tumor budding, we analyzed T1 colorectal carcinomas with immunohistochemistry, as the invasive front and tumor core can be studied on the same tissue sections (n=8 different tumors). The invasive front in these tumors can be easily identified with a pancytokeratin staining. High numbers of budding cells at the invasive front have been associated with poor prognosis and a tendency to metastasize [1, 2]. The invasive front, especially regions where budding cells were identified, showed a strong enrichment in CD68- and CD163-positive macrophages when compared to the tumor core (5,5-fold (*p*<0.001) and 3,5-fold (*p*= 0,0052 respectively; Figure 3D).

### Macrophages increase tumor “mesenchymal nature”

**Figure 4:**
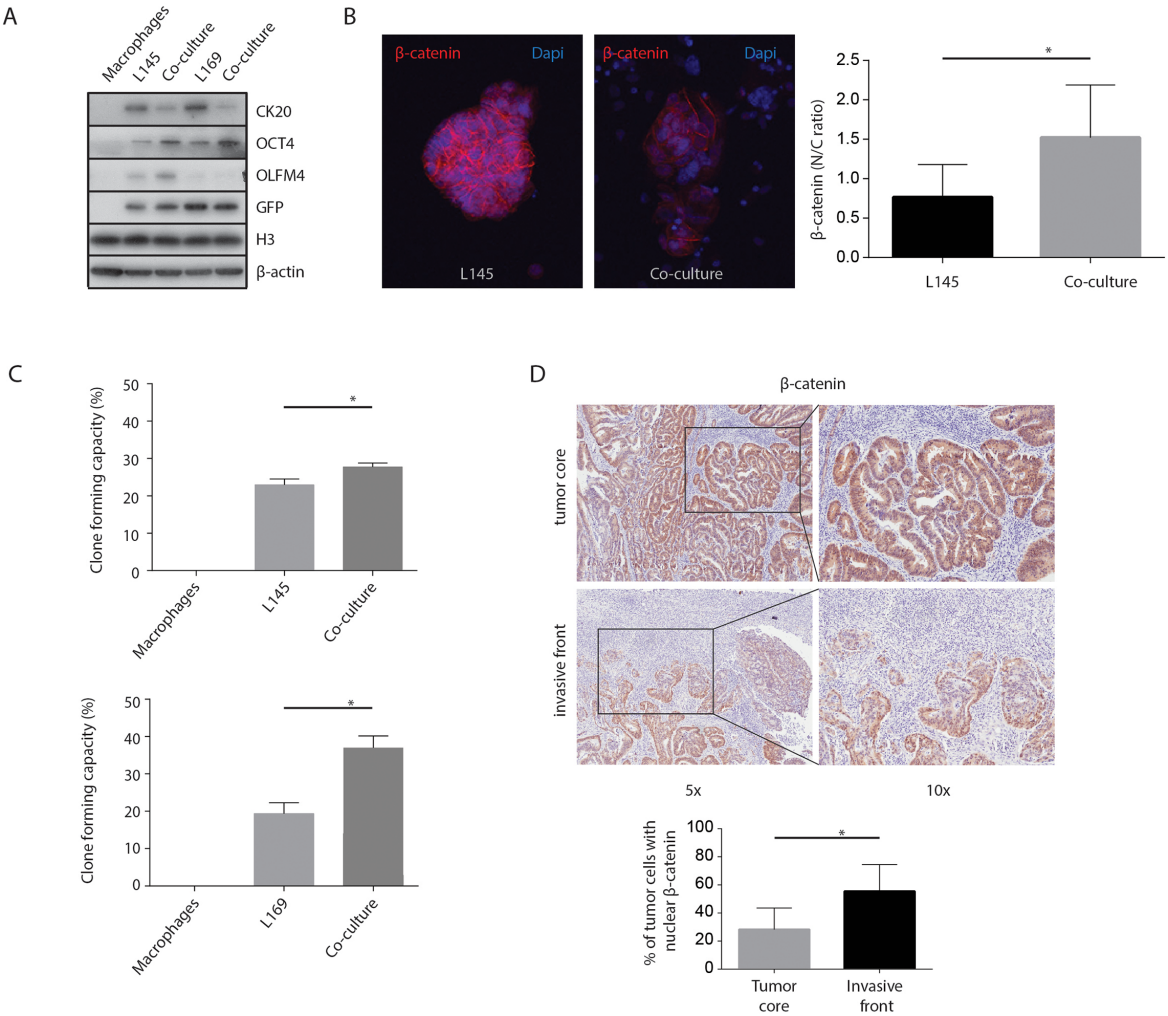
M2 macrophages increase tumor “mesenchymal nature” **(A)** Western Blots of whole cell lysates showing CK-20, OLFM4 and OCT4 expression, with GFP, H3 and β-actin as loading controls. **(B)** Representative confocal microscopy images of β-catenin, 20x magnification. The graph shows the quantification of the nuclear versus cytoplasmic β-catenin ratio measured in pancytokeratin positive cells. **(C)** Clone forming capacity assays for mono- and co-cultures. **(D)** Representative bright field microscopy images and quantification of immunohistochemical nuclear β-catenin staining in T1 carcinoma invasive front and tumor core, n=8 different tumors, one image field per tumor for the invasive field and one field for the tumor core; bar graphs show mean and SD, paired t-test, *p*=0.003.

An increase in stem cell capacity has been linked to colon cancer aggressiveness [12, 34]. Therefore, we tested whether macrophages can influence parameters of cancer differentiation. We found that macrophages induced increased expression of the stem cell markers OCT4 and OLFM4, and decreased expression of the differentiation marker CK-20 (Figure 4A). Furthermore, macrophages induced translocation of β-catenin from cell-cell junctions to the nucleus (Figure 4B). Finally, co-culturing with macrophages caused a significant increase in the regenerative, clone-forming, capacity of tumor cells (L145 p=0.002 & L169 p<0.001; Figure 4C). Macrophages alone did not form colonies. In addition, as several studies have found an accumulation of nuclear β-catenin at the invasive tumor front, while it remained membrane-bound at the non-invasive regions [35, 36], nuclear β-catenin was assessed by immunohistochemistry. When analyzing the expression of β-catenin in T1 colorectal carcinomas, immunohistochemistry showed increased nuclear β-catenin staining (almost 2-fold, *p*=0.003) in budding cells at the invasive front compared to the tumor core (Figure 4D). Together the results suggest that macrophages promote a more mesenchymal-like phenotype in nearby tumor cells.

### Macrophages induce loss of tight junction proteins at tumor cell-cell contacts

**Figure 5:**
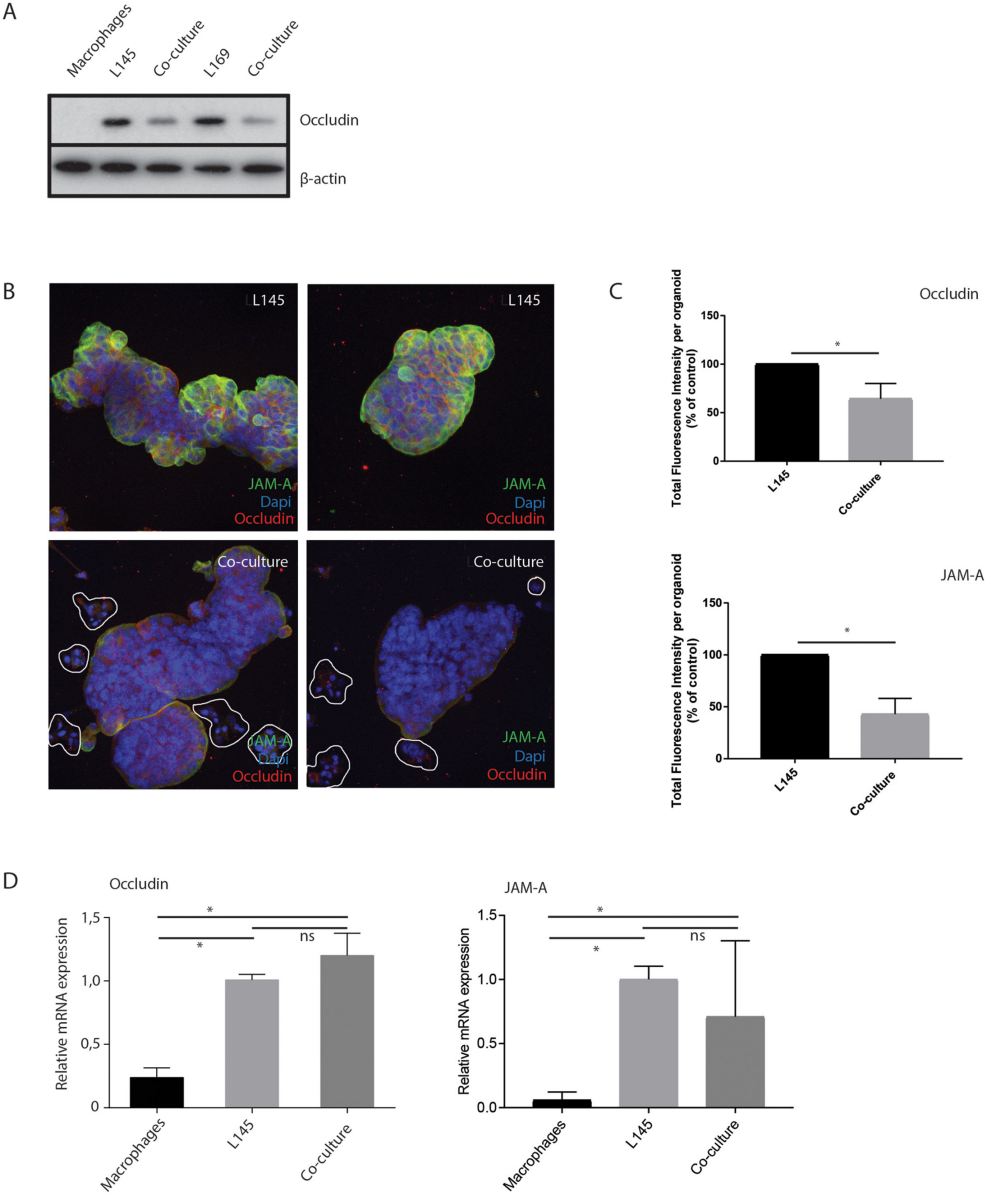
M2 macrophages induce loss of tight junction proteins at tumor cell-cell contacts **(A)** Western blot of whole cell lysates showing occludin expression in mono- and co-cultures with M2 macrophages. **(B)** Representative confocal microscopy pictures of immunofluorescence experiments for Occludin and JAM-A, 20 x magnification, n=7. Clusters of budding tumor cells are marked in the co-culture, **(C)** For each organoid, all the z-slides from the z-stack were added to allow quantification of fluorescence in all planes of the organoid, namely total fluorescence intensity per organoid. n=6 organoids for L145 and 8 organoids for the co-culture. Control L145 is set as 100 percent. **(D)** Relative mRNA expression of Occludin and JAM-A in mono- and co-cultures.

As tumor cell budding would necessitate cells separating from the bulk, this could indicate an effect on cell–cell junctions. Therefore, we examined whether tight junctions were affected during tumor cell budding. Western Blot analysis of total lysates showed a macrophage-induced decrease in the tight junction protein occludin (Figure 5A). Furthermore, immunofluorescence analysis confirmed an overall strongly reduced intensity of occludin and JAM-A levels between cancer cells (Figure 5B, 5C), suggesting reduced tight-junction mediated cell-cell adhesion. RT-qPCR analysis showed that macrophages did not affect occludin or JAM-A RNA levels (Figure 5D), possibly suggesting regulation at the (post-) translational level.

### MMPs control macrophage-induced loss of tight junction proteins and budding

**Figure 6:**
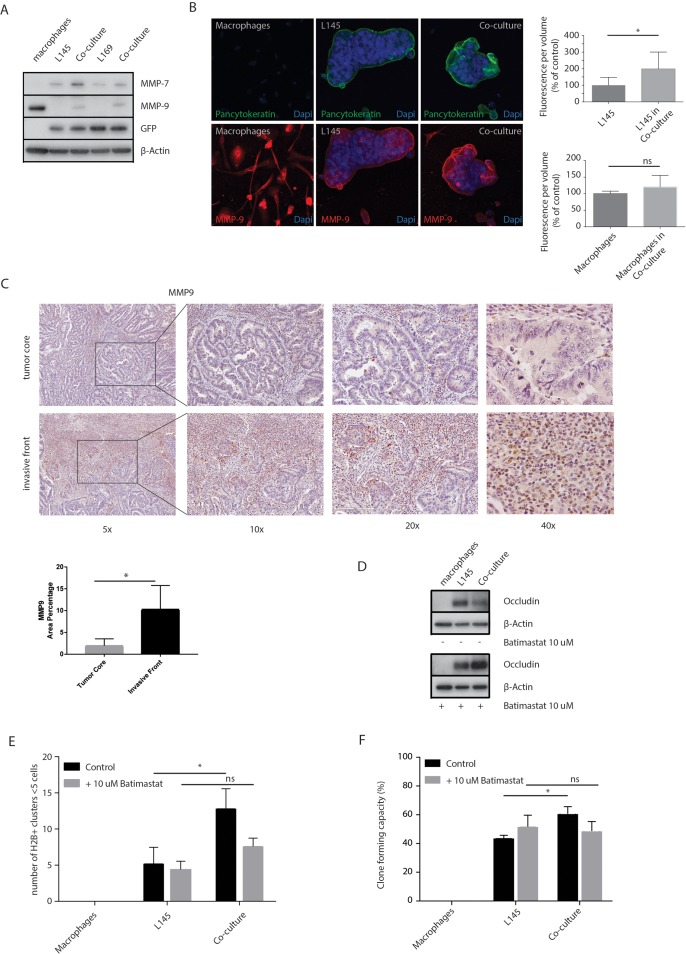
MMP inhibition prevents loss of tight junction protein expression and tumor cell budding **(A)** Western Blots of whole cell lysates showing MMP-7 and MMP-9 expression in mono- and co-cultures, with GFP and β-actin as loading controls. **(B)** Representative confocal microscopy images of MMP-9, 20 x magnification. The upper graph shows the quantification of MMP-9 measured in pancytokeratin positive cells, the tumor cells. The lower graph shows the quantification of MMP-9 measured in pancytokeratin negative cells, the macrophages. **(C)** Representative bright field microscopy images (5x, 10x, 20x and 40x) of immunohistochemical staining of MMP-9 expression at the invasive front and tumor core of T1 carcinomas, n=8 different tumors, one image field per tumor for the invasive field and one field for the tumor core; bar graphs show mean and SD, paired t-test, *p*=0.0116. **(D)** Western blots of whole cell lysates showing occludin expression in mono- and co-cultures with or without Batimastat 10 μM treatment for 48 hours. **(E)** Adherence assay showing the number of small cell clusters (≤5 cells) per condition with or without Batimastat 10 μM treatment for 48 hours, multiple t-tests, p<0.01. **(F)** Clone forming assay with or without Batimastat 10 μM treatment for 48 hours, two-way ANOVA, p<0.001.

MMPs are involved in extracellular matrix remodeling in physiological and pathological processes. Increased MMP activity can also contribute to the disassembly of intercellular junctions [37]. Western blot analysis of MMP-7 and MMP-9 expression showed that macrophages express MMP-9 but not MMP-7. Co-culturing macrophages with tumor cells caused increased expression of MMP-7 and MMP-9 in the tumor cells (Figure 6A-6B). Furthermore, an increase in MMP-9 expression was also seen around the budding cells at the invasive front of T1 colorectal carcinomas (Figure 6C). The percentage of area of MMP-9 staining was increased 5-fold (*p*=0.012) at the invasive front compared to the tumor core. These data show that the macrophage-MMP axis is likely to operate particularly in the invasive front of colorectal carcinomas. Next, we used Batimastat, an inhibitor of MMP activity, which binds the zinc ion in the active site of MMPs [38], to investigate a potential role for MMP activity in mediating the loss of tight junction proteins induced by macrophages. We found that Batimastat completely prevented macrophage-induced loss of occludin protein levels (Figure 6D). Strikingly, Batimastat also prevented macrophage-induced tumor cell budding and clone formation (Figure 6E, 6F).

### A potential role for NFκB in macrophage-induced tumor cell budding

**Figure 7:**
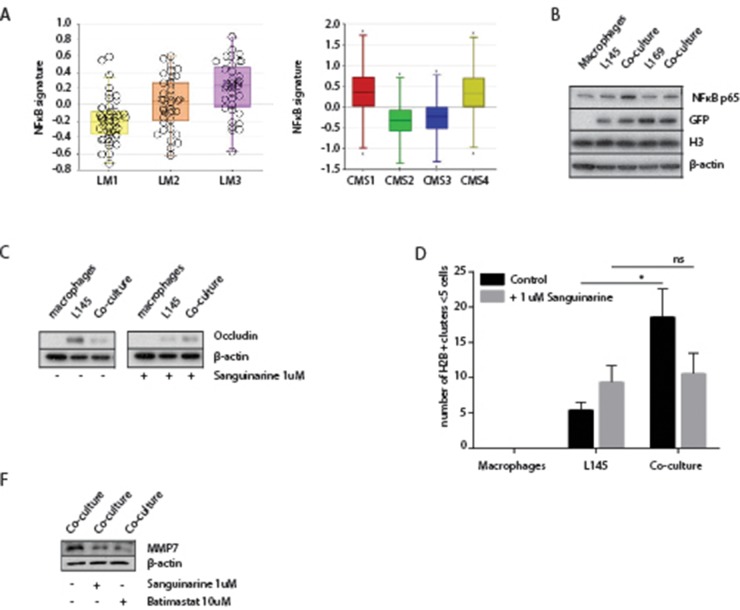
A potential role for NFκB in macrophage-induced tumor cell budding **(A)** Left box-whisker plot showing the gene signature expression of NFκB in the LM subgroups. Right box-whisker plot showing the gene signature expression of NFκB in primary CRC classified via the CMS-classification. **(B)** Western Blots of whole cell lysates showing NFκB p65 expression, with GFP, H3 and β-actin as loading controls. **(C)** Western blots of whole cell lysates showing occludin expression in mono- and co-cultures with or without Sanguinarine 1 μM treatment for 48 hours. **(D)** Adherence assay showing the number of small cell clusters (≤5 cells) per condition with or without Sanguinarine 1 μM treatment for 48 hours, multiple t-tests, p<0.01. **(F)** Western blots of whole cell lysates showing decreased MMP7 expression with Sanguinarine 1 μM treatment and Batimastat 10 μM, for 48 hours.

To further analyze the mechanism of macrophage-induced tumor cell budding we used the immune compendium macrophage signature to cluster several large tumor cohorts into subgroups (macrophage low-intermediate-high). We next identified the genes that were differentially expressed between macrophage-high and macrophage-low tumors. The promoters of these genes were subsequently analyzed for enrichment of specific transcription factor binding sites. This analysis revealed that NFκB-controlled genes were strongly enriched in macrophage-high tumors in all cohorts analyzed (Table 2). Moreover, a published NFκB target gene signature [39] was significantly higher expressed in the mesenchymal-like tumor subgroups LM3 and CMS4 (Figure 7A) and, as expected, in CMS1. Western blot analysis showed that NFκB p65 expression in tumor cells increased following co-culture with macrophages (Figure 7B). NFκB signaling facilitates malignant transformation of differentiated cells [40] and is high in the invasive front of human colorectal tumors [41]. Therefore, we hypothesized that NFκB signaling could play a role in the budding process induced by macrophages. To test this, we co-cultured tumor cells and macrophages in the presence or absence of the NFκB-inhibitor Sanguinarine [42]. Similar to the MMP inhibitor Batimastat, we found that Sanguinarine interfered with the decrease of occluding protein expression in the co-cultures as well as with tumor budding (Figure 7D). While the protein level of NFκB is not effected by either Sanguinarine or Batimastat (Supplementary Figure 5), the inhibition of NFκB by Sanguinarine resulted in a decrease in MMP7 in the co-culture (Figure 7F), suggesting a role for NFκB in the MMP-dependent decrease of Occludin. Surprisingly, batimastat also appeared to reduce the protein level of MMP7 (Figure 7F).

**Table 2:** NFκB target gene enrichment in Macrophage-high tumor subgroups

		Differentially expressed genes	NFκB rank		NFκB target genes			
Cohort	Groups	MΦ HIGH vs LOW	Total	Relative	UP in MΦ HIGH	UP in MΦ LOW	Fold	P (2×2 contingency)
AMC-90	3	1425	7/270	0.96	383	165	2,321212	3,00E-05
CIT-566	3	4515	8/355	0.98	524	257	2,038911	4,90E-07
LM-199	2	2348	1/128	1.0	266	112	2,375	2,30E-05
TCGA-286	3	4391	11/562	0.98	656	102	6,431373	1,70E-11

The tumors of the indicated cohorts were clustered into 2 or 3 groups using the macrophage signature of the immune compendium. Differentially expressed genes between MF-HIGH and MF-LOW subgroups were then identified in R2. Transcription factor binding site analysis of the differentially expressed genes in all cohorts revealed a strong enrichment for NFκB target genes in all MF-HIGH groups.

## DISCUSSION

In the present study we provide evidence that macrophages promote tumor cell budding in aggressive colon tumors through MMP-mediated degradation of tight junction proteins. The loss of tight junction integrity is commonly observed during oncogenic transformation. For instance, tight junction protein levels are decreased in colon cancer tissue when compared to normal intestinal tissue [43] and are inversely correlated with tumor grade [44]. The maintenance of tight junctions could play a pivotal role in the prevention of metastasis. Indeed, the budding phenomenon that we have observed in macrophage-tumor cell co-cultures reflects histological tumor budding, which is strongly correlated with poor prognosis [45]. Apart from destabilizing cell-cell interactions the loss of tight junctions also results in disturbed epithelial polarity which facilitates multidimensional tumor cell extrusion from the epithelial layer and promotes both tumor mass formation and invasion [46, 47].

Previous work has shown that paracrine chemokine signaling between tumor cells and macrophages can promote tumor cell migration in colon cancer [48, 49]. In T1-tumors there was a strong association between MMP-7 and nodal metastases in colon cancer [50]. The macrophage-MMP-tight junction-axis described in the present report adds a novel dimension to the functional interplay between both cell types in the tumor microenvironment. Occludin degradation has previously been implicated in the disruption of endothelial and epithelial barriers: High ROS levels in the endothelium of cerebral micro-capillaries resulted in occludin cleavage in the presence of high levels of MMPs [51]. Likewise, proteolytic cleavage of occludin by MMP-9 in the corneal epithelium disrupted barrier function through loss of tight junctions [52]. MMP-7 degrades laminin and type IV collagen to facilitate tumor invasion [53, 54]. However, MMPs play important roles in many different processes and their substrates are not limited to ECM components [55]. Although the relevant substrates for MMP-7 and MMP-9 in colorectal cancer progression are currently not known, meta-analyses have shown that high expression of either protease strongly predicts poor overall survival [56, 57]. Our work further connects high MMP-7 and MMP-9 expression to occludin degradation, loss of tight junctions and tumor cell budding in colon cancer. MMP inhibitors have been developed for anti-cancer therapy but have largely failed in the clinic due to unacceptable toxicity and lack of specificity and activity [58]. The design and pre-clinical testing of more specific MMP inhibitors [59] may re-open the way to targeting metastasis through MMP inhibition.

MMP-7 and MMP-9 are targets of the NFκB transcription factor [60–62]. We found a strong association of NFκB target gene expression with poor prognosis and with an aggressive macrophage-rich tumor phenotype. This is in line with studies showing that the activation of NFκB by prostaglandin E2 promotes [63] the formation of liver metastases and an increase in cancer stem cells [63]. NFκB also promotes tumor cell survival [64, 65] which may be especially relevant following detachment from neighboring cells. Factors that activate NFκB are over-expressed in tumor-macrophage co-cultures [66] Vice versa, inhibition of NFκB can prevent metastasis formation, either alone or in combination with 5-FU [64, 66, 67]. Together the studies point towards NFκB inhibition as a potentially effective strategy to suppress the budding phenotype and metastasis. The clinical development of NFκB inhibitors has been hampered by the central role that this transcription factor plays in innate and adaptive immunity, precluding long-term inhibition of NFκB for therapeutic purposes.

Most targeted therapies have been developed against tumor cell-intrinsic pathways. However, stroma-targeting therapies have also been developed, including anti-angiogenic therapy. The VEGF-targeting antibody bevacizumab is now part of the standard treatment of metastatic colorectal cancer. More recently, the TAM has received attention as a *bona fide* target in the tumor microenvironment. Pre-clinical work in glioblastoma multiforme models has shown that macrophage ‘re-education’ through CSF1R inhibition caused re-polarization of TAMs towards an M1 phenotype, which dramatically increased survival, and regressed established tumors [68]. Unfortunately, in our experiments the CSF1R inhibitor BLZ-945 did not prevent macrophage-induced tumor cell budding, nor did it induce tumor cell killing (KT, unpublished results). Nevertheless, targeting TAMs through alternative pathways remains an attractive option for developing new treatment strategies against aggressive colon cancer.

So far, the molecular classification of human colon cancer has been limited to primary tumors [3–9]. We provide evidence for the existence of a CMS4-like liver metastasis subtype (LM3). Both CMS4 and LM3 show high expression of mesenchymal genes indicating a high stromal content [11, 17] and/or a more mesenchymal nature of the neoplastic cells [30]. In addition, both subtypes show high expression of the macrophage signature and both subtypes are associated with a reduced survival probability. A further understanding of the pro-tumorigenic influence of macrophages on tumor progression may therefore be relevant for both early, local disease, and for metastatic cancer.

In conclusion, our study has identified a novel macrophage-initiated NFκB-MMP pathway that causes loss of tight junction integrity and tumor budding. The co-culture system described here may serve as a new model system to investigate tumor budding in more detail, including for instance the influence of various cytokine/chemokine pathways, matrix components and additional stromal cell types. Such work should lead to the identification of potentially targetable pathways in the treatment of aggressive colon cancer subtypes.

## MATERIALS AND METHODS

### Cell culture

Monocyte-derived macrophages (MDMs) were prepared as previously described [69]. In brief, PBMC were isolated from venous blood obtained from adult healthy donors by gradient centrifugation using Ficoll-Paque Plus (GE Healthcare, Uppsala, Sweden). Monocytes were isolated from the PBMC suspension by gradient centrifugation using Percoll® (GE Healthcare, Uppsala, Sweden). The cells were taken up in RPMI 1640 medium containing 1% FCS, 1% Ultraglutamine and 1% PenStrep with a density of 0.35×10^6^cells/ml. After 1 hour of adhering, the non-adherent cells were washed away and the remaining monocytes were cultured in RPMI 1640 medium containing 10% FCS, 1% Ultraglutamine and 1% PenStrep and stimulated with M-CSF (R&D Systems, Minneapolis, USA) 25 ng/ml for 8 days. For further polarization of the macrophages to a M2 phenotype, stimulation with IL-10 (Peprotech, Rocky Hill, USA) 10 ng/ml 48 hours followed. For generating M1 macrophages, monocytes were isolated as mentioned above and stimulated with GM-CSF (R&D Systems, Minneapolis, USA) 25 ng/ml for 10 days.

Patient-derived colonosphere lines were established as described before [33]. The colonospheres are cultured in non-adherent 10 cm dishes in Stem Cell Medium with 10 ng/mL b-FGF (Abcam), which is refreshed twice a week. All cell culture was carried out at 37°C in a 5% CO_2_ humidified incubator.

In Figure 3A, fluorescent coculture images were binarized by using the Thresholding automatic setting ‘Huang’ by Huang & Wang (1995) [70] in Fiji/ImageJ for all three images.

### Bioinformatics analyses

We used our previously generated dataset containing gene expression profiles of 119 liver metastases deposited at Array Access E-TABM-1112 and we used a dataset containing gene expression profiles of 3232 primary CRC described before [9]. These datasets were uploaded into the R2 Genomics analysis and visualization platform (http://r2.amc.nl) for the various types of analyses described in the text and figure legends.

### Western blot

Lysates were prepared either in RAS lysis buffer (20 mmol/L HEPES pH 7.4, 1% Nonidet P-40, 150 mmol/L NaCl, 5 mmol/L MgCl_2_, and 10% glycerol) or in Leamli buffer (10% glycerol, 2% SDS, 63 mM Tris-HCl pH 6.8). Nuclear proteins were extracted with nuclear extraction buffer (25mM HEPES pH 7.4, 500mM NaCl, 5mM MgCl_2_, 1mM DTT, 0.2% NP-40, 10% Glycerol, Protease/Phosphatase inhibitors). Equal amounts of protein were loaded on NuPAGE Novex Bis-Tris Mini Gel (Invitrogen) and were analyzed by Western blotting.

### Immunofluorescence

Cells were harvested and fixed in PBS containing 4% of formaldehyde and permeabilized with ice-cold (−20°C) methanol. Cells were blocked in PBS containing 0.1% Tween and 5% BSA; cells were incubated with primary antibodies Pancytokeratin (MA5-13156, Pierce Antibodies, 1:100 dilution), β-catenin (9582, Cell Signaling, 1:100 dilution), Occludin (33-1500, Life Technologies, 1:100 dilution), JAM-A (sc-25629, Santa Cruz, 1:100 dilution), MMP-9 (ab38898, Abcam, 1:100 dilution) overnight at 4°C. Cells were subsequently washed and incubated with secondary antibodies (goat anti-rabbit Alexa Fluor568, 1:500 dilution, and goat anti-mouse Alexa Fluor647, 1:500 dilution; Invitrogen) for 1 hour at room temperature. DAPI (0.5 μg/mL) was used to stain the nuclei. Prolong gold was added to preserve the fluorescent signal and to fix the slides. Pictures were analyzed with Imaris software.

### Immunohistochemistry

Slides were created by transverse sectioning (4μm). The paraffin-embedded slides were deparaffinated with xylene and rehydrated through a series of ethanol concentrations. Endogenous peroxidase activity was blocked by incubating in 0.3% H_2_O_2_ in methanol at room temperature for half an hour, after which antigen retrieval was achieved by heating the slides in a citrate buffer, pH 6.0, for 20 minutes; followed by cooling in the same buffer for 20 minutes. The slides were incubated with a diluted primary antibody overnight at 4°C or for 1 hour at room temperature. Incubation with an undiluted secondary antibody (Brightvision Poly-HRP®) for 30 minutes followed. Rinsing between steps was performed with PBS. The slides were developed with diaminobenzidine and counterstained with Mayers’ hematoxylin. Hereafter, the slides were dehydrated and mounted with cover slips. The following primary antibodies were used: CD68 (333801, Biolegend,1:800 dilution, 1hr incubation), CD163 (321101, Biolegend, 1:400 dilution, 1hr incubation), Pancytokeratin (MA5-13156, Pierce Antibodies, 1:500 dilution, 1hr incubation), β-catenin (9582, Cell Signaling, 1:75 dilution, 1hr incubation), MMP-9 (ab38898, Abcam, 1:50 dilution, overnight at 4°C incubation). Positive and negative controls of these stainings are shown in Supplementary Figure 3.

Slides were digitized and analyzed via Aperio ImageScope or ImageJ/Fiji when appropriate. For the immunohistochemical stainings of CD68, CD163 and MMP9, ImageJ/Fiji was used to separate out the DAB staining using the Color Deconvolution application (Vector: H DAB was used; Examples of this are shown in Supplementary Figure 4. Then, the Auto Threshold plugin was applied to binarize all images. The Auto Threshold setting used was “Yen” from Yen et al. (1995) [71] and was applied to all images to keep the thresholding identical. The area percentage of these binarized areas was calculated by ImageJ/Fiji and used to quantify these stainings. The immunohistochemical staining of β-catenin was done by manually counting the total number of nuclei and the β-catenin positive nuclei.

### FACS

The expression of a panel of cell surface markers was analyzed using a FACS Calibur (BD Biosciences, San Diego, USA). After harvesting the cells, antibody incubation steps were carried out at 4°C for 30 min in PBS + 1% BSA + 0.1% sodium azide + 1% rabbit serum. Dead cells were excluded using viability marker SYTOX (ThermoFisher). Antibodies used were CD68-FITC (EBM11, DAKO, Denmark), CD86-PE (Clone IT2.2, BD Biosciences), CD163-PERCP-CY5.5 (clone GHI/61, Biolegend, San Diego, USA), CD14 APC-AF750 (clone RMO52, Beckman Coulter, Brea, USA), CD206-PC-7 (clone 3.29B1.10, Beckman Coulter) and CD16-APC (clone 3G8, Life technologies, Frederick, USA).

For FACS-based cell cycle analysis, cells were incubated for 30 minutes with 1μM BrdU at 37°C. BrdU-positive cells were detected with an anti-BrdU-FITC antibody (BD Bioscience) according to the manufacturer's protocol. For determination of DNA content 10μg/ml propidium iodide (PI) was added in the presence of 250 μg/ml RNase.

### Colony forming assay

Harvested cells were made single cell via trypsinization, washed with PBS and were filtered through a 40-μm cell strainer. Cells were plated in Matrigel in a concentration of 100 GFP+ cells/well, Medium was refreshed twice a week. Two weeks after plating, GFP+-colonospheres were counted.

### RT-qPCR

Total RNA was isolated according to the manufacturer's protocol (RNeasy Mini Kit, Qiagen) from colonosphere cell lines. cDNA was synthesized from 500 ng of total RNA using iScript cDNA Synthesis Kit (Bio-Rad Laboratories). Next, cDNA was diluted 20-fold and 5 μl was used for cDNA amplification. The amplification was performed in an iCyclerthermocycler (Bio-Rad Laboratories) using iQ SYBR Green Supermix (Bio-Rad Laboratories). Occludin primer sequence: Forward, 5′-AAGAAGCCTA-3′ and Reverse, 5′-TTGGAGCCATCC-3′. JAM-A Primer sequence: Forward, 5′-ACACCACCAGACTCGTTTGC-3′ and Reverse, 5′-GACCTTGACCTCCCCATAGC-3′, Housekeeping gene B2M: Forward, 5′-GAGGCTATCCAGCGTACTCCA-3′ and Reverse, 5′-CGGCAGGCATACTCATCTTTT-3′ mRNA expression levels were quantified using iCycler software (Bio-Rad Laboratories) and were normalized to *B2M*.

### Statistical analysis

All statistical analyses were performed using SPSS 23.0 software (IBM SPSS, Chicago, IL, USA) or GraphPad Prism version 6.00 for Windows (GraphPad Software, San Diego California USA, www.graphpad.com). The Pearson chi-square test was used to compare differences in discrete or categorical data. Continuous data were tested for normality with d’Agostino &Pearson normality test. Normally-distributed data were analyzed with t-test or one-way ANOVA. If data would not have been normally distributed, analysis by means of the Mann–Whitney U test or Kruskal–Wallis test would have been performed. DFS and OS curves were generated using the Kaplan–Meier method; differences between survival curves were assessed by log rank test. A level of P <.05 was considered statistically significant.

## SUPPLEMENTARY MATERIALS FIGURES


