# Supplementary material for: Macrophages induce “budding” in aggressive human colon cancer subtypes by protease-mediated disruption of tight junctions
Source: Oncotarget. 2018 Apr 13;9(28):19490–507. doi: 10.18632/oncotarget.24626 (PMC5929404; doi:10.18632/oncotarget.24626)
Supplement: Supplementary file 1 [file oncotarget-09-19490-s001.pdf]

Macrophages induce "budding" in aggressive human colon cancer subtypes by protease-mediated disruption of tight junctions

SUPPLEMENTARY MATERIALS

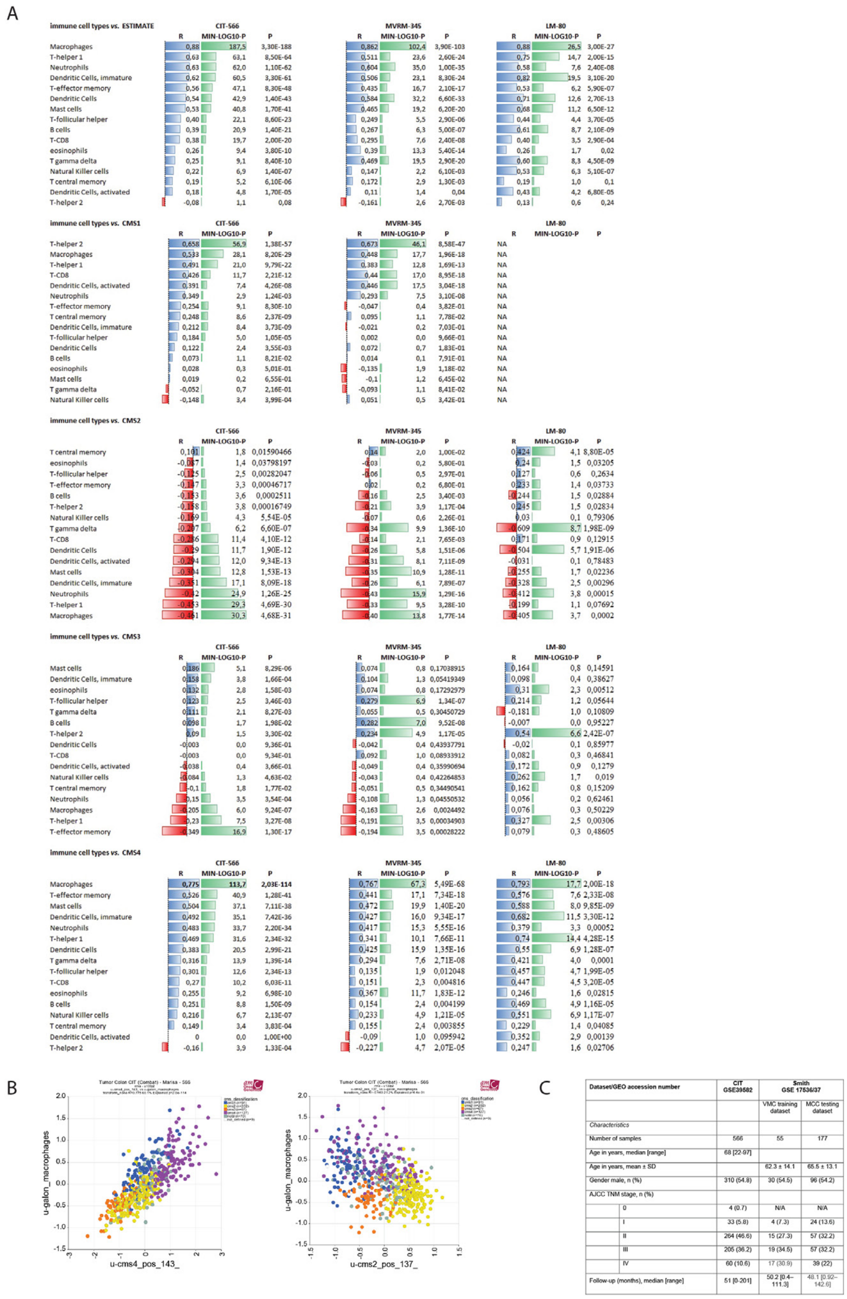

C

Dataset/GEO accession number		CIT GSE39582	Smith GSE 17536/37	
			VMC training dataset	MCC testing dataset
Characteristics				
Number of samples		566	55	177
Age in years, median [range]		68 [22-97]		
Age in years, mean ± SD			62.3 ± 14.1	65.5 ± 13.1
Gender male, n (%)		310 (54.8)	30 (54.5)	96 (54.2)
AJCC TNM stage, n (%)				
	0	4 (0.7)	N/A	N/A
	I	33 (5.8)	4 (7.3)	24 (13.6)
	II	264 (46.6)	15 (27.3)	57 (32.2)
	III	205 (36.2)	19 (34.5)	57 (32.2)
	IV	60 (10.6)	17 (30.9)	39 (22)
Follow-up (months), median [range]		51 [0-201]	50.2 [0.4–111.3]	48.1 [0.92–142.6]

Supplementary Figure 1: Immune cell gene signatures in CMS subtypes. (A) For each immune cell gene signature, the meta-gene values were generated and stored as a separate track. These tracks were subsequently compared 1 by 1 in exactly the same manner as one would compare the expression of two genes in a tumor cohort. One way analysis of variance (ANOVA) was then used to assess significance of the correlation and xy plots were generated for all track-track correlations. An overview of the correlation R-values belonging to each xy-plot is shown, accompanied by p-values (–LOG10-transforemd). **(B)** Examples of the correlation xy-plots for the macrophage gene signature in CMS4 (strong positive correlation) and CMS2 (strong negative correlation). The CMS subtypes are color-coded as indicated in the legend. **C)** Characteristics of the CIT-566 and Smith-232 cohorts.

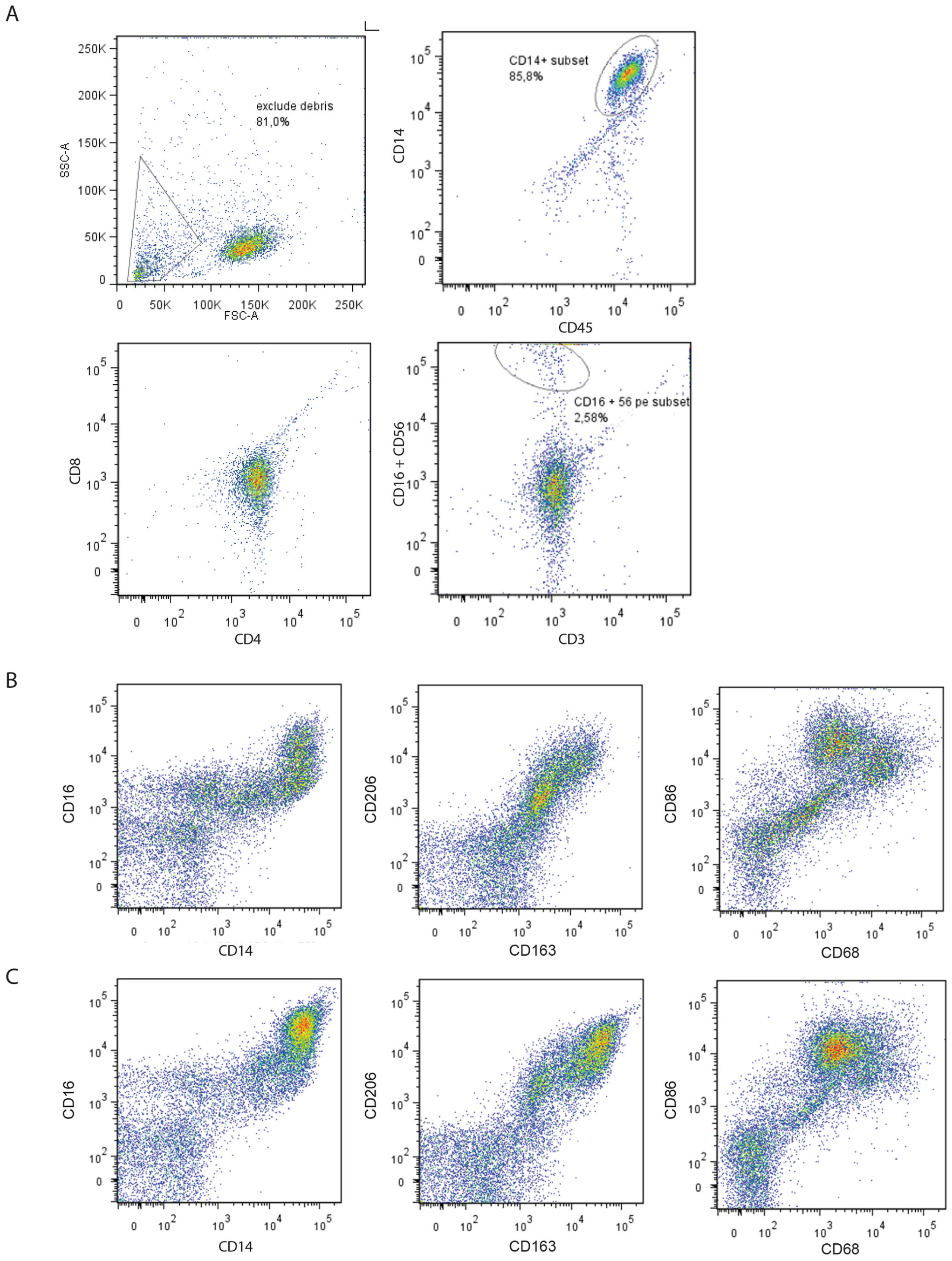

Supplementary Figure 2: Macrophage polarization with IL-10. (A) Isolation of CD14 positive monocytes from PBMCs. **(B)** CD-marker expression after 8 days of M-CSF stimulation. **(C)** CD-marker expression after 8 days of M-CSF stimulation and 48 hours of IL-10 stimulation.

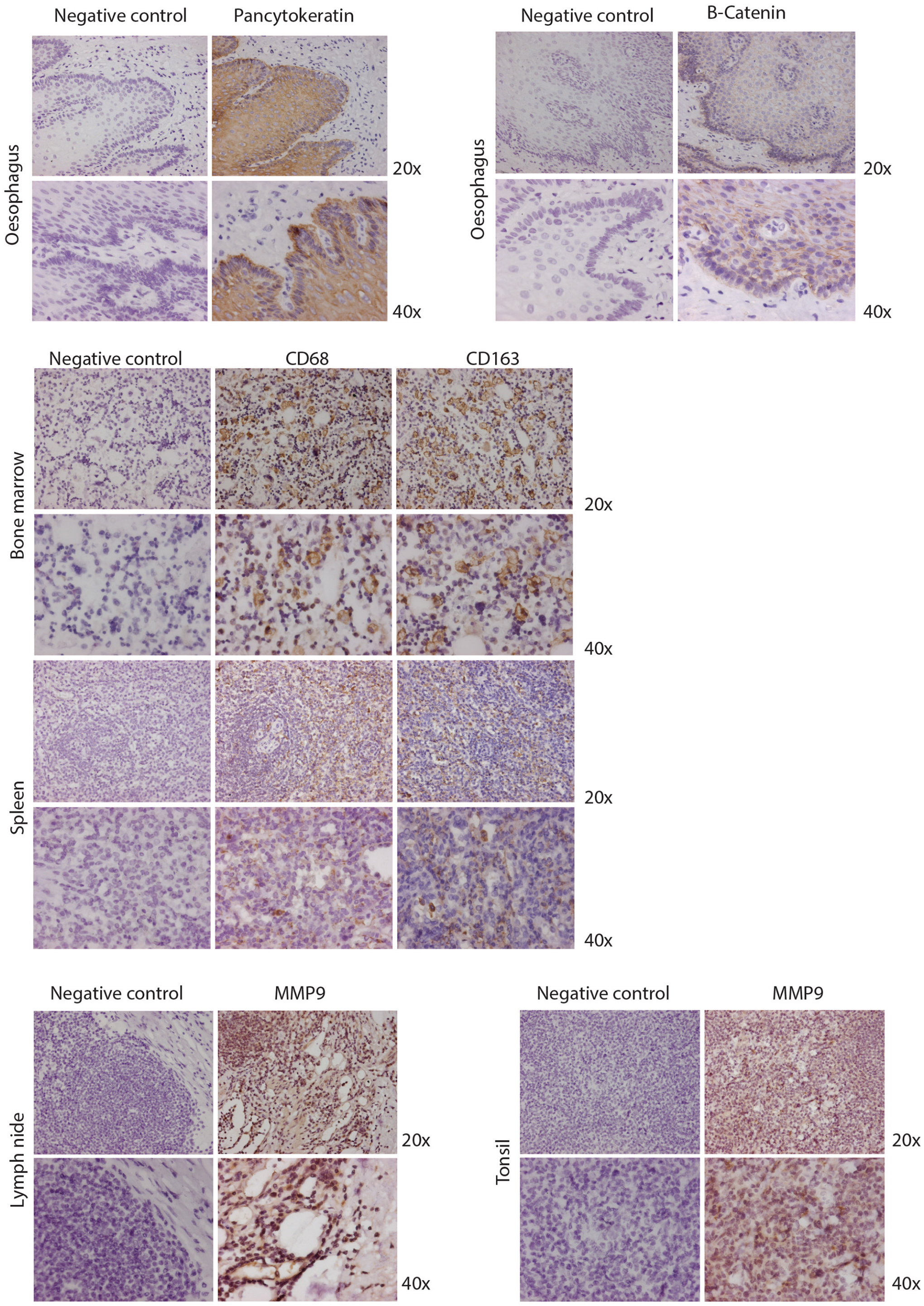

Supplementary Figure 3: Positive and negative controls immunohistochemical stainings. Overview of the negative and positive controls for the immunohistochemistry stainings of Pancytokeratin, b-Catenin, CD68 and CD163, 20x and 40x magnification.

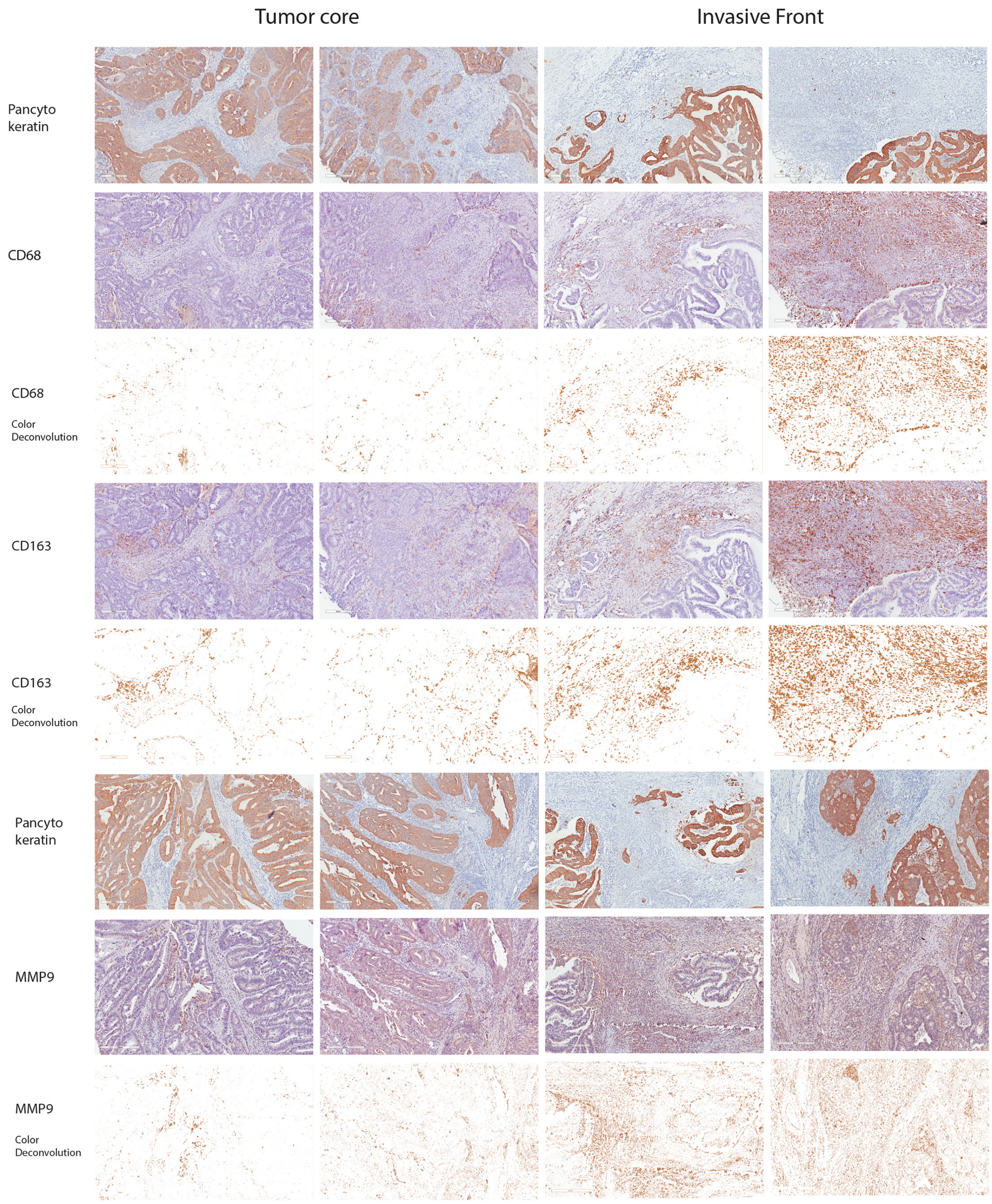

Supplementary Figure 4: Immunohistochemical stainings - Color Deconvolution Method. Examples of stainings where the image program Fiji/ImageJ was used for Color Deconvolution (Vector: H DAB). All images are a 5x magnification.

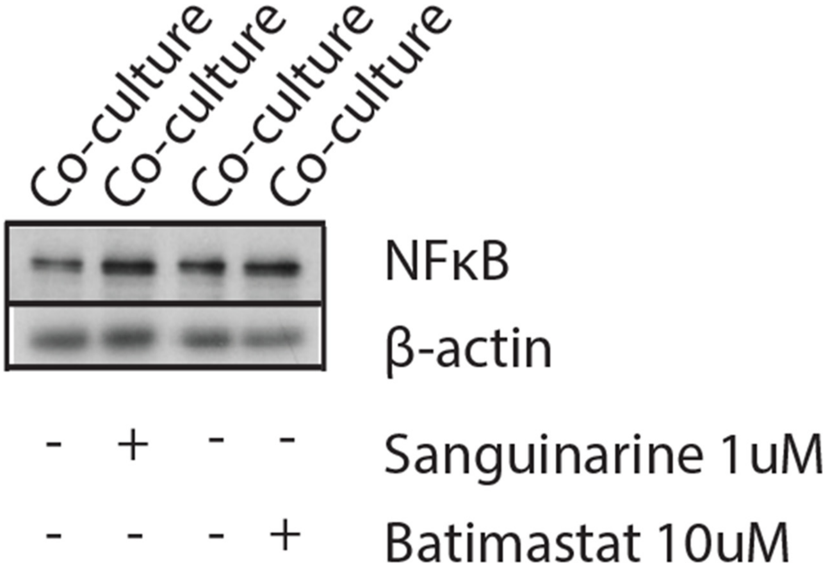

Supplementary Figure 5: M2 + L145 Co-culture Protein levels NFκB. Western blot showing co-culture protein levels of NFκB unaffected by treatment with Sanguinarine 1uM and Batimastat 10uM for 48 hours.
